# Working memory guidance of visual attention to threat in offenders

**DOI:** 10.1371/journal.pone.0261882

**Published:** 2022-01-07

**Authors:** Tamara S. Satmarean, Elizabeth Milne, Richard Rowe

**Affiliations:** Department of Psychology, University of Sheffield, Sheffield, United Kingdom; University of South Florida, UNITED STATES

## Abstract

Aggression and trait anger have been linked to attentional biases toward angry faces and attribution of hostile intent in ambiguous social situations. Memory and emotion play a crucial role in social-cognitive models of aggression but their mechanisms of influence are not fully understood. Combining a memory task and a visual search task, this study investigated the guidance of attention allocation toward naturalistic face targets during visual search by visual working memory (WM) templates in 113 participants who self-reported having served a custodial sentence. Searches were faster when angry faces were held in working memory regardless of the emotional valence of the visual search target. Higher aggression and trait anger predicted increased working memory modulated attentional bias. These results are consistent with the Social-Information Processing model, demonstrating that internal representations bias attention allocation to threat and that the bias is linked to aggression and trait anger.

## Introduction

Antisocial behaviour (ASB) encompasses a wide range of traits and behaviours with substantial negative consequences for both victims and offenders [1]. ASB comes at a high cost, varying as a function of harm caused to the victim, to property, to the community, and costs incurred within the criminal justice system [2]. These impacts highlight the importance of ASB reduction [3]. Recent violence reduction and prevention practices have increasingly drawn on social-cognitive models of aggression and violent behaviour [4, 5]. These models propose a sequence of information processing steps involved in social interaction. Cognitive biases (reliably distorted representations of some aspect of reality) [6] may facilitate ASB [7] at each step. Interventions may target bias reduction at specific steps to reduce ASB.

The Social Information-Processing model (SIP, [8]) has been used extensively to understand the role of cognition in aggressive social interactions. The model proposes five steps: (1) Cue Encoding, (2) Cue interpretation, (3) Choice and clarification of goals, (4) Response construction or selection, (5) Response decision [8]. Aggressive individuals are hypothesized to exhibit both early and late cognitive biases in terms of hypervigilance to threatening stimuli and increased attribution of hostile intent in socially ambiguous situations, termed “hostile attribution bias” [9, 10]. For example, if someone spills their drink on you, a hostile attribution could be that the person purposefully tried to harm or embarrass you whereas a nonthreatening attribution would be that it was accidental. These processing deficits are hypothesized to stabilise throughout development to predict a pervasive aggressive response bias [11, 12].

Distinct patterns of information processing from encoding [13] to behavioural enactment [14] can be observed in individuals displaying higher aggression [15]. Specific patterns may underlie different forms of aggression [16], associated behavioural outcomes [17], and distinct biases in social information processing. For example, reactive aggression as a response to perceived provocation [9] has been linked to hypervigilance to and difficulties in disengaging from threat during encoding and intent attribution. Proactive aggression, planned to achieve specific goals, has been linked to construction and selection of aggressive responses to ambiguous situations [10, 18]. Similarly, trait anger has been associated with cognitive biases [19, 20] and is likely to influence social information processing.

In order to target interventions to specific information processing steps, it is necessary to understand the underlying cognitive mechanisms. Processing in the SIP model is hypothesised to be guided by emotion and a “database” encompassing memory, social schemas, acquired rules, and affect-event links [10]. Emotion and memory are therefore at the core of the SIP framework [21]. As a transition resource for mental structures (such as behavioural schemas and scripts), working memory (WM) is directly involved in real-time processing [10]. However, the mechanisms through which WM influences information processing stages have not been elucidated. It is therefore necessary to examine WM biases of visual perception in the context of the SIP model.

WM templates as forms of internal representations or internal goals [22], precede information selection and processing [23]. For example, everyday goal directed visual search tasks (e.g., searching for a face in a crowd) are typically preceded by a cue, such as knowing that the search target has dark hair or specific facial features. This cue in turn leads to the creation of an attentional template for the search goal (i.e., finding the face matching the description), which guides the visual search to match the attentional template [24]. Such a template helps to manage the overwhelming amount of information available in the environment by providing top-down guidance of attention allocation to memory-matching, task-relevant stimuli [25]. WM guidance of perception can become disadvantageous when cognitive resources are allocated toward threatening stimuli as distractors [26] matching WM templates [27].

Biased WM guidance of attention may contribute to a tendency to over allocate attention to threatening stimuli. Trait anxiety has been linked to attentional biases toward threat [28]. Similarly, aggression has been linked to an increased attentional bias towards hostile stimuli [13]. However, aggression related attentional bias research has addressed the possibility of attention allocation being guided by aggressive internal representations. Yao et al [22] found that highly anxious participants showed an increased attentional bias toward threatening faces when holding an angry face in mind. WM guidance of attention has not been explored in aggression.

Using a combined WM and visual search task, this study examines the extent to which WM modulated attentional bias in a former offender sample. There is a higher incidence of aggression-related knowledge structures (i.e., schemas, internal representations) in offender populations [29] shown to account for almost a quarter of variance in aggression scores [30]. Internal representations held in WM affect encoding, processing, and integration of information [31]. Given this guidance effect [32, 33], we expected social stimuli held in WM as templates to constrain allocation of cognitive resources to stimuli matching internal representations. Additionally, participants were expected to preferentially encode [34] and maintain angry faces in WM [35] thereby tending to allocate cognitive resources toward angry or hostile stimuli (i.e., an anger superiority effect). Lastly, we explored unmapped links between WM modulations of visual attention and later stages in social information processing. In line with the SIP model [36] an increased bias toward potentially threatening stimuli was expected to be predicted by higher aggression, trait anger, and hostile attribution bias.

## Method

The method, data preparation protocol, and data analysis plan were pre-registered with the Open Science Framework (OSF, https://osf.io/chjpz/?view_only=9366ad5cc34d425a9f4de533257402e2). This study was approved by the University of Sheffield Ethics Committee. Written informed consent was obtained and the data were analysed anonymously.

### Participants

Power analysis assuming a small to medium effect size of d = 0.40 (for the expected main effect of congruency (H1)- in line with findings from Yao et al [22]) showed that a sample size of 70 was recommended to achieve power of .95 (α = 0.05, one-tailed). As participants with less than 70% correct trials on the visual task were excluded from the final analysis, participants were recruited beyond the recommended sample size (N = 131) to compensate for potential exclusions. Following exclusions, data from 113 participants were analysed (M_age_ = 41.29, SD_age_ = 12.23; 33.63% female). In the final sample, 85% of the participants were White, 7% were Black, and the remaining 8% reported mixed, Asian, or other ethnic backgrounds. With RTs to the dual task as DV, the power levels afforded (α = 0.05, one-tailed) for the expected interaction (congruency and emotional valence of the WM template) in the current sample size for a small (f = 0.10), medium (f = 0.25), and large (f = 0.40) [37] effect were .18, .75, and .99 respectively. The power level afforded to detect a medium effect size f = 0.15 for the composite reactive aggression and anger variable and hostile attribution in the current sample was .85. Participants were recruited via Prolific (www.prolific.co) on the basis of being English speakers, based in the United States or United Kingdom, and answered “Yes” to the custom screening: “Have you ever been in prison for committing a crime?” offered by Prolific.

### Measures

***Social information processing patterns*** were measured using an abbreviated version of the Social Emotional Information Processing Questionnaire (SEIP-Q) [15]. SEIP-Q variables were assessed in relation to vignettes comprising ambiguous scenarios in which Person A is the victim of Person B’s adverse action. The vignettes were designed to contain either direct aggression (e.g., physical aggression) or relational aggression (e.g., rejection). Participants were required to identify with Person A. Attribution was assessed using four Likert-scale questions measuring: direct hostile intent (e.g., “This person wanted to damage my car”), indirect hostile intent (e.g., “This person wanted me to feel unimportant”), instrumental non-hostile intent (e.g., “This person was in a hurry to get in to work”), and benign intent (e.g., “This person scratched my car by accident and didn’t notice”). Negative Emotional Response was measured using two Likert-scaled questions (e.g., How likely is it that you would be angry if this happened to you”). Participants were asked how likely they would be to enact a behavioural response that is either socially appropriate, overtly aggressive or relationally aggressive. SEIP-Q scores for each construct were averaged on the same 0–3 scale. The SEIP-Q has been validated in community and clinical samples with good to excellent reliability and validity [15]. The scale assesses all stages detailed in Crick and Dodge’s [8] SIP model except encoding. In this sample, alpha coefficients were strong for hostile attribution (α = 0.87) and negative emotional response (α = 0.79) but lower for benign attribution (α = 0.47) and instrumental attribution (α = 0.56).

***Reactive and Proactive aggression*** were measured using the Reactive-Proactive Aggression Questionnaire (RPQ) [38] which contains 23 items measuring proactive (12 items) and reactive aggression (11 items) based on frequency of behaviours on a 3-point Likert scale which ranges from 0 (never) to 2 (often). Example reactive aggression items include “Gotten angry when frustrated” and “Reacted angrily when provoked by others”, whereas proactive aggression items include “Vandalized something for fun” and “Hurt others to win a game”. The RPQ has been validated in adolescent and adult populations [Spielberger 1999], has demonstrated high reliability and internal consistency (Cronbach’s alpha > 0.81) and can differentiate between community and forensic samples [39].

***Trait Anger*** was measured using the 10-item Trait Anger subscale of the State-Trait Anger Expression Inventory–II [40]. Items were scored from 1 (“Almost never”) to 4 (“Almost always”). Trait anger correlates with behavioural aggression [41] and has demonstrated high reliability and validity (including concurrent validity) across clinical and non-clinical samples [42].

#### Dual task

**Fig 1:**
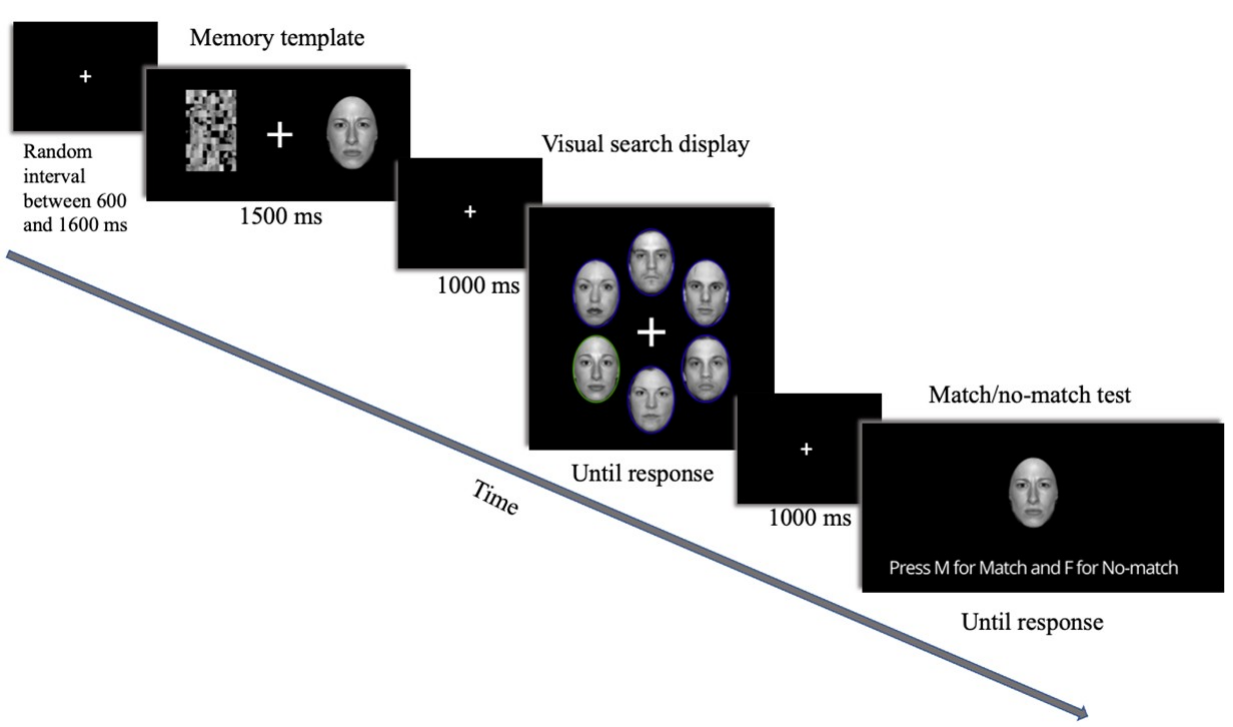
Dual task trial example.

The Gorilla Experiment Builder (www.gorilla.sc) was used to collect online reaction time data. Following Burra et al [43], attentional bias to threat was inferred from reaction times (RTs) on a dual task, consisting of a working memory task and a visual search task. As depicted in Fig 1, a memory template consisting of an angry or neutral face oval was initially presented lateral to a fixation point. The position opposite the face oval contained a scrambled version of the face oval, balancing the visual display [26]. This was followed by a visual search array consisting of six faces (target and five other neutral faces, 3 male and 3 female faces, identities and gender randomly allocated in the display). Following the visual search array, participants were presented with a match/no-match test display and asked whether a face matched the memory template displayed at the beginning of the trial. The target was always presented laterally (never at vertical midline) and consisted of a face oval surrounded by an unfilled oval (coloured oval shape having a width of 20 pixels) whose colour was distinct from the remaining 5 ovals. The unfilled colour oval surrounding the target varied randomly between green (with the remaining neutral faces in the visual display being surrounded by blue) and blue (with the remaining neutral faces in the visual display being surrounded by green). The memory template was identical in valence and identity to the target for half of the trials (congruent condition) and had the same identity and a different emotional valence from the target for the other half (incongruent condition). The remaining neutral faces in each visual display were randomly extracted from the pool of neutral face ovals (excluding the target) and a random list generator assigned visual search display positions. Participants were required to memorize the face in the memory template, identify the gender of the target in the visual search display, and identify whether the face in the match/no-match test was the same as the memory cue. Further detail on the development and validation of the dual task can be found in S1 File.

#### Stimuli

Task stimuli for both WM and visual search tasks comprised angry and neutral faces (6 male, 6 female) selected from the NimStim stimulus set [44]. Following previous visual search studies faces with visible teeth were discarded, selected face ovals were cropped at or near the hairline, and all pictures were converted to grey-scale [43]. Given that pictures of faces are prone to low-level confounds, likely to influence visual search performance, the contrast and luminance histograms of the pictures were equalized using the SHINE Matlab toolbox [45]. Each face oval took up 20% of the screen height and 14% of width. Each face oval and scrambled counterpart in the memory template was placed 5% of the screen away from the fixation cross and face ovals in the visual search displays were placed 10% of the screen away from the fixation cross.

### Procedure

Each trial began with a white fixation point on a black background, presented for a random interval between 600 and 1600 ms [43]. Participants were presented with the memory template for 1500 ms which they were asked to memorise [22]. This was followed by a fixation point displayed for 1000 ms which was subsequently replaced by the visual search array, displayed until response. Participants were required to identify the target, which was surrounded by a colour singleton distinct to the colour surrounding the distractors and report the target face gender as quickly and accurately as possible. Participants were instructed to use their left index finger to press “F” if the face oval is female and the right index finger to press “M” for male face ovals. In each trial, the visual search display was followed by a fixation cross for 1000 ms and a match/ no-match test whereby a face oval was presented at the centre of the screen and the participants were required to answer whether the face matched the memory template by pressing the “F” key for “no-match” and the “M” key for “match”. Before the experiment, participants completed 20 practice trials with on-screen feedback. If fewer than 14 face genders (70%) were correctly identified during practice (identification of the gender of the target), the participant was asked to read the instructions for the task again and complete another 15 practice trials. Each participant completed 192 trials in two blocks of 96. Participants completed the dual task followed by the self-report measures in the order listed above.

### Data preparation

Data preparation and planned analyses were pre-registered with the OSF. Although callous-unemotional traits were included in the pre-registration, differential patterns of WM modulated attention allocation associated with callous-unemotional traits are beyond the scope of this paper. Additionally, high correlations were found between self-reported aggression, anger, and SEIP-Q response enactment variables (see S1 Table). As we pre-registered hypotheses for the relationships between hostile attribution, anger, aggression, and WM biases of visual perception, hostile attribution was added as a predictor in regression models with aggression and anger as predictors. The extended pre-registered exploration of links between WM modulations of visual attention and patterns of social information processing is included in S2 File (Tables 7, 8, and 9).

Analyses were performed using RStudio 4.0.2 [46]. As planned, outliers were Winsorized; values outside 1.5 interquartile ranges from the Tukey Hinges (lower and upper hinge corresponding to the first and upper quartiles or 25^th^ and 75^th^ percentiles respectively) were rescaled to the last valid value within the range. This approach improves score reliability across a range of attentional bias task modifications [47]. There were no missing trials in the combined visual task. Following Burra and Kerzel [34], participants with < 70% correct trials on either visual search or match/no-match test were excluded from analysis (n = 18). Incorrect responses on both visual search and match/no-match were removed from the dataset analyses [48]. Following Burra et al [43], RTs under 200 ms were deemed unlikely to reflect genuine responses and were therefore removed from the combined visual task dataset. RTs on the visual search task and the match/no-match test were extracted and analysed separately.

Missing values for the self-report measures were inspected using heat maps. No participants were missing > 20% of questions within a self-report measure. Examination of histograms plots, box plots, and z scores for non-normality indicated that proactive aggression was significantly positively skewed. These scores were converted to a binary variable which distinguished at 0 from scores above 0 (coded “1”). Response enactment scores for directly aggressive responses were positively skewed which was removed by square root transformation.

#### Computation and reliability of attentional bias data

Bias scores (BS) were computed by subtracting mean RTs/ experimental condition of interest from mean RTs for congruent and neutral trials (see Table 1). Using 5000 random splits, Spearman-Brown corrected reliability estimates found low-to-medium split-half reliabilities of the BS. Alternative trial-level bias scores [49] were computed following the pre-registered analysis plan. Using the weighted trial method, a time-series of trial- level BS per participant was produced by subtracting the RTs for each trial from the weighted mean of all trials of the opposite type (i.e., baseline trials comprising congruent and neutral trials). Table 1 shows that split-half reliabilities for trial-level BS were considerably higher than the reliabilities of the BS, trial-level BS were henceforward used as measurement of attentional bias, and were referred to as BS (bias scores). BS_*POSITIVE*_ (mean of *positive* trial-level BS, indicating attentional bias toward target stimuli) and *BS*_*NEGATIVE*_ (mean of *negative* trial-level BS, indicating attentional bias away from target stimuli) scores were calculated for each of the three conditions above.

**Table 1:** Bias scores: Computation and reliability.

*Bias score and computation*	*Spearman-Brown (r*_*SB*_ *) for traditional BS*	*Spearman-Brown (r*_*SB*_ *) for Trial-Level BS*
BS1: Comparing RT_congruent& neutral_ to RT_congruent& angry_	*0*.*24*	*0*.*81*
BS2: Comparing RT_congruent&neutral_ to RT_incongruent&angry_	*0*.*43*	*0*.*86*

BS, Bias Scores.

BS for neutral targets identified whilst holding an angry face in WM (BS2_*POSITIVE*_) were highly correlated to the remaining BS and were excluded from further analyses. Regression models having the excluded BS as DV did not have any significant predictors beyond the predictors for retained BS which are summarised below. BS1scores, indicating attentional bias toward and away from angry targets identified whilst holding an angry face in WM (second and third hypotheses) and the BS2_*NEGATIVE*_ scores (second and third hypotheses), indicating an attentional bias away from neutral targets identified whilst holding an angry face in mind were retained.

### Analysis plan

#### I. Do WM templates guide visual search for naturalistic faces and is there an effect of the emotional valence of the WM templates?

A two-way repeated measures ANOVA with RTs (latency) to targets in visual search displays as the dependent variable and trial congruency (whether the emotional valence of the WM template matched that of the target, i.e., congruent vs incongruent) and emotional valence of the WM template (angry vs neutral) as independent variables was expected to reveal an attentional bias toward congruent compared to incongruent stimuli. An interaction effect was also expected, whereby participants would display faster RTs to trials in which both WM templates and targets consist of angry faces (congruent & angry) compared to trials in which WM templates and targets consist of neutral faces (congruent & neutral).

#### II. Are WM visual selection biases predicted by self-reported aggression and trait anger?

Individuals displaying higher aggression and trait anger scores holding an angry face template in WM were expected to display an attentional bias toward a matching angry target and be slower to find (or demonstrate a bias away from) a neutral target preceded by an angry attentional template. This effect was expected to be specific to angry faces and therefore absent when attentional templates were neutral. Hierarchical regressions were planned to evaluate the prediction of attentional bias (BS1 and BS2 as DVs), from aggression subfactors and trait anger (as predictors). Reactive aggression and trait anger scores were significantly correlated (r = 0.64). Similarly, response enactment for relationally and directly aggressive behavioural responses were significantly correlated (r = 0.63). Composite variables were created by averaging reactive aggression and trait anger for the former and response enactment scores for the latter. Alpha coefficients for the composite variables were excellent (α = 0.88 for the anger/reactive aggression composite and α = 0.87 for the response enactment for aggressive responses composite). We found significant correlations between anger, aggression, and SEIP-Q variables. Consequently, the pre-registered relationship between hostile attribution and bias scores was investigated separately, within the regression models with anger and aggression as predictor variables. This enabled the examination of the relationship between aggression, anger, and BS before controlling for hostile attribution. The hierarchical regression model predicting BS added age, gender, and education at step 1. Composite reactive aggression and trait anger scores were added at step 2. Proactive aggression was added at step 3 and hostile attribution was added at step 4.

## Results

### I. Do WM templates guide visual search for naturalistic faces and is there an effect of the emotional valence of the WM templates?

**Fig 2:**
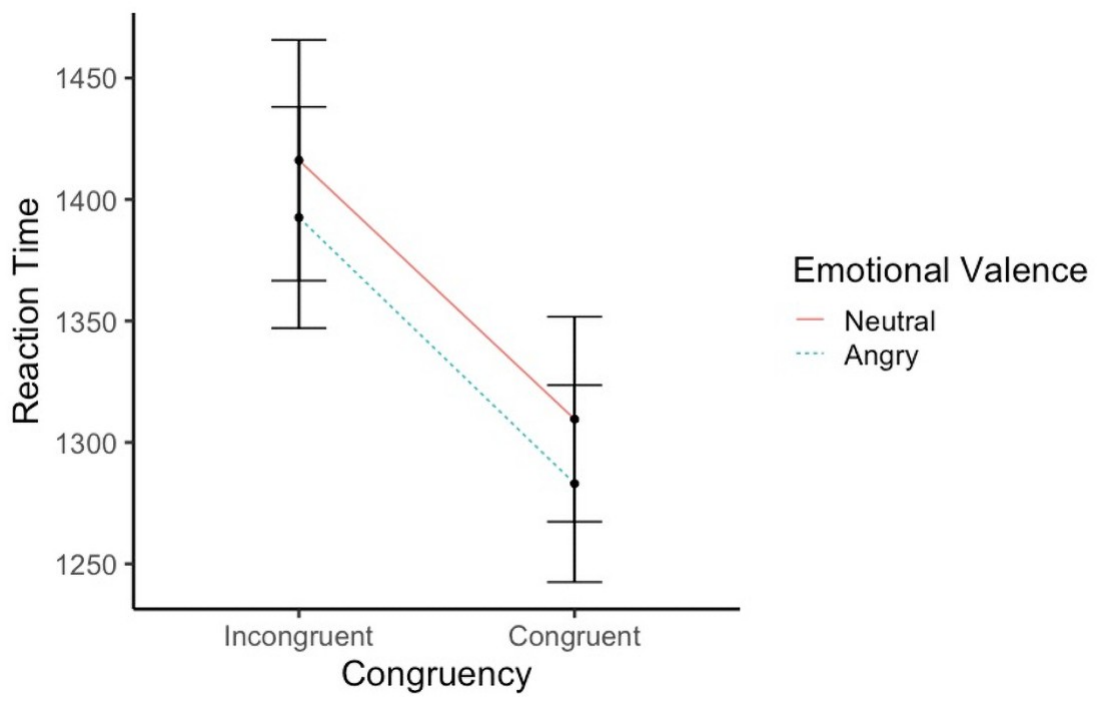
Changes in reaction times as a function of congruency and emotional value.

As expected, the ANOVA showed a main effect of congruency (*F*(1, 112) = 75.7, *p<*0.001, *η*_*p*_^*2*^ *=* 0.39) with faster RTs to congruent (M = 1296.3, SD = 438.92) than incongruent trials (M = 1404.07, SD = 504.84). A further main effect of emotional valence of the memory template was found (*F*(1, 112) = 6.68, *p =* 0.01, *η*_*p*_^*2*^ = 0.05) with faster RTs when visual search displays were preceded by angry memory templates (M = 1337.82, SD = 460.46) rather than neutral memory templates (M = 1362.85, SD = 490.96). Contrary to the hypothesis, there was no interaction between congruency and emotional valence of the WM template (*F*(1, 112) = 0.02, *p =* 0.87, see Fig 2).

### II. Are WM visual selection biases predicted by self-reported aggression, trait anger, and hostile attribution?

Hierarchical regression modelling is summarised in Tables 2–4. Composite reactive aggression and anger positively predicted attentional bias toward angry faces whilst holding an angry face in WM (BS1_POSITIVE_, *β =* 0.22, *p =* 0.03, model at step 2) and negatively predicted attentional bias away from angry faces whilst holding an angry face in WM (BS1_NEGATIVE_, *β =* -0.19, *p =* 0.05). Reactive aggression and anger scores did not predict bias towards or away from targets after proactive aggression was added. There were no significant anger or aggression predictors of BS2_*NEGATIVE*_ scores; however, hostile attribution scores were a significant negative predictor of attentional bias away from neutral faces identified whilst holding an angry face in mind (BS2_*NEGATIVE*_, β = -0.21, p = 0.04, model at step 4).

**Table 2:** Hierarchical multiple linear regression analysis predicting attentional bias toward angry faces whilst holding an angry face in WM (BS1_POSITIVE_).

	*β*	*SE*	*p*	*R*^*2*^_*Total/Change*_
*Step 1*				0.003
Gender	-0.08	0.10	0.41	
Education	0.06	0.10	0.53	
Age	-0.14	0.10	0.15	
*Step 2*				0.04/0.028*
Gender	-0.03	0.09	0.75	
Education	0.06	0.09	0.52	
Age	-0.07	0.10	0.48	
Reactive aggression and anger	0.22	0.10	0.03*	
*Step 3*				0.04/0.29
Gender	-0.03	0.10	0.71	
Education	0.06	0.09	0.51	
Age	-0.07	0.10	0.48	
Reactive aggression and anger	0.17	0.11	0.11	
Proactive Aggression	0.11	0.10	0.29	
*Step 4*				0.05/ 0.20
Gender	-0.03	0.09	0.77	
Education	0.04	0.09	0.72	
Age	-0.07	0.10	0.47	
Reactive aggression and anger	0.14	0.11	0.20	
Proactive Aggression	0.11	0.10	0.30	
Hostile Attribution	0.12	0.10	0.20	

*β*, standardized regression coefficient.

*p < .05.

**Table 3:** Hierarchical multiple linear regression analysis predicting attentional bias away from angry faces whilst holding an angry face in WM (BS1_NEGATIVE_).

	*β*	*SE*	*p*	*R*^*2*^_*Total/Change*_
*Step 1*				0.03
Gender	0.15	0.09	0.10	
Education	-0.02	0.09	0.84	
Age	0.19	0.10	0.05	
*Step 2*				0.06/0.05*
Gender	0.11	0.10	0.25	
Education	-0.01	0.09	0.84	
Age	0.12	0.10	0.22	
Reactive aggression and anger	-0.19	0.10	0.05*	
*Step 3*				0.05/0.41
Gender	0.11	0.10	0.24	
Education	-0.006	0.09	0.95	
Age	0.13	0.10	0.21	
Reactive aggression and anger	-0.16	0.11	0.14	
Proactive Aggression	-0.09	0.10	0.41	
*Step 4*				0.06/0.17
Gender	0.12	0.10	0.23	
Education	-0.008	0.09	0.93	
Age	0.12	0.10	0.22	
Reactive aggression and anger	-0.17	0.11	0.12	
Proactive Aggression	-0.09	0.10	0.34	
Hostile Attribution	-0.13	0.10	0.17	

*β*, standardized regression coefficient.

*p < .05.

**Table 4:** Hierarchical multiple linear regression analysis predicting attentional bias away from neutral faces whilst holding an angry face in WM (BS2_*NEGATIVE*_).

	*β*	*SE*	*p*	*R*^*2*^_*Total/Change*_
*Step 1*				0.02
Gender	0.05	0.09	0.54	
Education	-0.06	0.09	0.50	
Age	0.04	0.09	0.64	
*Step 2*				-0.009/0.18
Gender	0.03	0.10	0.78	
Education	-0.06	0.10	0.50	
Age	0.001	0.10	0.99	
Reactive aggression and anger	-0.14	0.10	0.18	
*Step 3*				-0.006/0.24
Gender	0.03	0.10	0.76	
Education	-0.05	0.10	0.63	
Age	0.004	0.10	0.97	
Reactive aggression and anger	-0.09	0.11	0.44	
Proactive Aggression	-0.12	0.11	0.24	
*Step 4*				-0.01/0.03*
Gender	0.02	0.10	0.82	
Education	-0.03	0.10	0.75	
Age	0.00	0.10	0.99	
Reactive aggression and anger	-0.03	0.11	0.76	
Proactive Aggression	-0.12	0.10	0.25	
Hostile Attribution	-0.21	0.10	0.04*	

*β*, standardized regression coefficient.

*p < .05.

### III. Match/no-match test accuracy: Exploratory analysis

As a proxy measure of the quality of WM templates, match/no-match test accuracy (measured as percentage of correct trials) was introduced as a DV in a multiple linear regression having emotional valence of the memory template (angry vs neutral) and congruency (congruent vs incongruent) as predictors. Emotional valence positively predicted accuracy (β = 0.45, p< 0.01) with higher accuracy for angry (*M =* 82.72, *SD =* 13.64) compared to neutral faces (*M =* 76.49, *SD =* 15.7).

## Discussion

This study examined how emotional faces as WM templates biased visual search and whether this bias was predicted by self-reported aggression, trait anger, and social-emotional information processing variables. As expected, WM templates guided attention during visual search for naturalistic face targets. Increased aggression and trait anger predicted increased WM attention bias. Bias was further linked to self-report measures and key constructs in social information processing, thus tapping into a previously unexplored link between WM templates and SIP stages. The combined visual task utilised in this study has not been employed in previous research; thus, the results build on the attention as well as the antisocial behaviour literature to spotlight specific relationships between cognitive biases and measures of aggression and trait anger.

### Do WM templates guide visual search for naturalistic faces and is there an effect of the emotional valence of the WM templates?

This study examined the effect of emotional faces as WM templates on visual search. Consistent with predictions, faster reaction times were found for congruent visual search targets (i.e., targets identical to the templates held in WM), demonstrating a direct effect of emotional faces held in WM on visual search. Similar effects of congruency, whereby participants respond faster to targets sharing features with WM templates, have been found in previous studies examining WM biases of visual perception [50], including in forensic populations [51]. The present study was the first to extend this effect to naturalistic, emotional faces. Moving beyond low-level stimuli and feature based search (i.e., shapes and lines) [52], current findings provide further evidence that faces are likely to be encoded as integrated representations. That is, the faces presented in the visual search display and as WM templates were grayscale ovals and luminance and contrast were averaged across stimuli used in the task, thereby reducing the effectiveness of potential feature-based searches [53]. Consequently, the WM template (or internal representation) was likely encoded and maintained in WM as a single object which effectively guided visual search.

Following the SIP model [54], we expected that visual search for an angry face whilst holding an angry face in WM (i.e., a negatively valenced internal representation) would be more efficient than visual search for a neutral face whilst holding a neutral face in mind. We found no supporting evidence for this prediction. There was however an effect of emotional valence of the WM template on visual search. Specifically, when participants held an angry face in WM, they responded to targets in visual search significantly faster than when holding a neutral face in WM, regardless of the emotional valence of the target. Participants were also significantly more likely to remember angry over neutral faces for the match/no-match test, suggesting a partial processing advantage for emotional compared to neutral faces.

Evidence of an anger superiority effect has been found across multiple populations including undergraduates [54, 55], community samples [34], anxious [56, 57] and aggressive populations [58]. In this study, the preferential encoding and maintenance of aggressive faces into WM was followed by an enhanced performance for all targets within the visual search task. Drawing on the SIP model, the findings demonstrate an overall WM bias of visual perception by emotional faces; however, the effect was not limited to angry faces as expected. Instead, an emotional (here negatively valenced) WM template appeared to broadly (and positively) bias visual search, increasing effectiveness and distractor suppression during visual search. In other words, holding a negative internal representation enabled participants to meet task goals effectively.

### Are WM biases of visual selection predicted by self-reported aggression, trait anger, and hostile attribution?

As expected, an increase in reactive aggression and anger (composite score) predicted an increased attentional bias towards angry targets identified whilst holding an angry template in WM. Lower aggression and anger scores predicted increased avoidance of angry faces whilst holding an angry face in WM. Trait anger [19] and aggression [59] have been linked to attentional biases in previous research. This study further showed that variation in aggression and trait anger also predict WM modulated biases of visual search. In line with the SIP model [8], reactive aggression and trait anger scores did not predict increased attentional bias to angry compared to neutral faces when proactive aggression was controlled. Although the SIP further postulated a link between reactive aggression and hostile attribution [10] there was no evidence for this association in the present research. As previously suggested by Oostermeijer et al [60], these findings indicate SIP mechanisms may not be differentiated for aggression subtypes, which can exert an influence on both early and late steps in social information processing. A lower hostile attribution bias was associated with avoidance of neutral targets when holding an angry face in WM. These findings are consistent with the SIP model [61], specifically that increased aggressiveness and hostile attribution predicted increased WM modulated attentional bias towards emotional (here angry) and away from neutral faces respectively.

Behavioural studies have demonstrated robust WM biases of visual search using low-level stimuli (e.g., shapes and lines) [62]. However, in a social environment, social content must be held in WM to facilitate social exchanges [22]. Moreover, WM is fundamental to effective communication and is particularly involved in social interactions when decoding and interpreting others’ emotions and intentions [63]. Angry faces are recognised more accurately [64], preferentially encoded [55] and maintained in working memory [35]. This study constitutes a first step in understanding the effect emotional faces held in WM have on visual search, suggesting that the bias toward emotional faces is amplified by trait anger and aggression whereas lower aggression, anger, and hostile attribution predicted avoidance of targets. We found angry templates were preferentially encoded and maintained in WM compared to neutral. In line with models conceptualising WM as a common resource, dynamically distributed according to prioritisation of salient stimuli [65], our findings indicate a greater proportion of cognitive resources was drawn to emotional faces. Further, holding an angry face in WM resulted in enhanced processing of both angry and neutral targets. This broad bias may be specific to goal-directed visual search for emotional naturalistic stimuli.

Threat detection is fundamental to survival. According to the threat capture hypothesis, threatening stimuli are automatically detected, employing early mechanisms which are independent of cognitive control [66]. Soto et al [67] found that an attentional template held in WM can guide “early parts of the search process in an involuntary manner” (p. 260). The WM bias of visual attention found here may reflect a WM guided involuntary deployment of cognitive resources to the detection of an emotionally valenced target in a social environment which would match the WM template. However, Burra and Kerzel [34] demonstrated that attentional biases to threat are only partially automatic and dependent upon context and task demands. Indeed, the main effect of congruency found in this study, with neutral targets found faster when holding a neutral attentional template in WM, supports the hypothesis that top-down attentional biases can extend to processing neutral stimuli when required by task demands. Furthermore, from a SIP perspective, it may be that holding an emotionally valenced internal representation- in this case, an angry face- makes detecting a matching emotion in a social environment task-relevant. Consequently, visual search would be increasingly efficient which may explain our observed enhanced visual search performance whilst holding an angry face in WM. This study focussed on angry and neutral WM templates and targets. Future research should investigate the effect of angry faces in relation to other emotions as well as neural events corresponding to these effects using the contralateral delay activity, an event-related potential component indicative of working memory maintenance [26].

Within the SIP model, higher aggression is associated with a higher prevalence of biased (negative) internal representations expected to guide attentional resources towards threatening stimuli and increase attribution of hostile intent during ambiguous social interactions [10]. In a recent paper, a neural activity boost was found during maintenance of self-associated (compared to other-associated) information in WM, indicative of prioritization by top-down attention [68]. This supports the above link between aggression and bias within the SIP, namely, that individuals displaying higher aggression or trait anger are more likely to allocate disproportional amounts of cognitive resources to hostile stimuli which are viewed as threatening.

In this study, the WM modulated bias of visual perception was further associated with hostile attribution, linking top-down biases of attention to interpretation of intent. Hostile attribution was positively linked to anger but not to reactive aggression. Further links to anger and later stages in the SIP are detailed in S1 Table. Hostility biases, referring to the tendency to attribute hostility in social interactions have been linked to perceptual biases [69] and aggression [70], pointing toward a general hostility bias mechanism Smeijers et al [12]. Our findings support the hypothesized links between hostile attribution and the proposed SIP model core that consists of acquired rules, memory, and social schemas. Within the model proposed by Smeijers et al [12], SIP stages are mapped onto the hierarchical Gaussian filter [71] and tenets of the free-energy principle [72]. In other words, memories, rules, and social schemas form complex blueprints of social interaction, optimized to reduce uncertainty about the world. For example, a person who has repeatedly experienced a certain ambiguous social situation as resulting in an aggressive outcome (e.g., a heated argument ending in a fight) has adjusted their belief and will display a general tendency to attend to and interpret specific cues as indicating similarities between the given situation and the blueprint.

This line of inquiry leads to two important considerations. The first refers to the acquisition and reinforcement of the information at the core of the SIP model. Individuals displaying higher anger and aggression also report higher anger-rumination tendencies [73]. These comprise rehearsing and dwelling upon hostile information which are believed to reinforce tendencies toward anger and aggression [74]. In the present study we have addressed how an induced representation can bias visual selection. That increased aggression and trait anger predicted an increased bias toward emotional faces could also be explained by an increased tendency toward anger-rumination. The latter could facilitate encoding of hostile cues, leading to an increased focus on hostile information held in WM and consequently to a stronger modulation of visual selection. In the context of the SIP, increased rumination would also lead to increased overall selectivity for hostile cues and reinforcement of hostile biases.

The second consideration refers to the applicability of a general hostility tendency to other sensory modalities and stimulus categories. Previous research has linked aggression and trait anger to biases toward semantic threat. For instance, individuals displaying higher anger tendencies were slower to disengage from hostile words [75] and exhibited difficulties in processing nonhostile information in ambiguously hostile visual scenes [76]. Using neuroimaging data and an emotional word Stroop task, [77] found that attentional bias for antisocial words predicted aggression and that this relationship was fully mediated by amygdala reactivity to angry faces. The present study has also demonstrated a relationship between WM modulated attentional bias and hostile attribution bias. Overall, these findings provide support for a database of memory and schemas guiding cognitive processes across sensory modalities [24] whilst demonstrating the relative advantage of stimuli likely to be present and form part of a social interaction. That is, patterns of mediation may be completely different for neural activation to angry faces when using auditory semantic antisocial stimuli (i.e., direct provocation) compared to read words (i.e., inferred threat).

### Limitations

The findings should be considered in the context of some limitations. When preparing the task stimuli, we aimed to reduce low-level confounding effects (e.g., contrast, luminance [45]) which may have reduced ecological validity. Using natural faces as stimuli may have added noise due to physical differences. This concern was mitigated by presenting perceptually balanced real faces, demonstrating that WM modulated attentional bias was not due to low-level confounds, as proposed elsewhere [78]. Additionally, physical differences in naturalistic faces are present in daily interactions and their removal from experimental designs (e.g., by using schematic drawings of faces, [79]) results in a penurious summary of the allocation of cognitive resources during social interactions.

The present research further found significant relationships across variables of interest, good-to-excellent reliability for attentional bias scores, and self-reported measures showed reliability indices comparable to previous studies. Although later stages of the SIP model have typically been investigated using vignettes to illustrate ambiguous social scenarios and questionnaires to assess potential emotional and behavioural responses [12], future studies should seek to reproduce these findings in paradigms with higher ecological validity, i.e. measurement of responses to ongoing provocation. Composite scores were used in this study due to high correlations between trait anger and reactive aggression as well as between response enactment scores for relationally and directly aggressive responses (see S2 File). Whilst the composite variables had high reliabilities, these findings raise the issue of considerable conceptual overlap between established measures of trait anger and aggression, and between aggression subtypes in the context of social information processing stages.

Collecting reaction times data over the internet is a potential limitation of this study as the experiment was completed across a range of devices and visual display settings. Online data collection has increased considerably in the last decade and the quality of such data, albeit requiring careful examination [80], has been found to be acceptable [81, 82]. Moreover, results were compatible with previous studies using similar paradigms. Online data collection enabled recruitment of a wide range of former offenders. However, the participants’ criminal history and specifically whether they have a history of violence is unknown. Also, the lack of a control group means that no inferences can be made regarding whether these results are specific to a population with an incarceration history. Thus, future replication in distractor free laboratory-based experiments and extension to non-offender and offense-specific samples would be valuable.

The task used in this study, which combined a WM and a visual search task, the identity of the WM template and that of the target were always the same, providing an overlap between WM content and targets of visual attention. Given that RTs were measured in relation to participants’ identification of the gender of the target, it was possible that participants reacted to the gender of the face held in WM, therefore bypassing the visual search aspect of the task. However, the task was cognitively demanding and there was no evidence of participants having bypassed the visual search component to react to the gender of the WM representation (i.e., a pattern of learning in the RT data). RTs would become faster with practice as participants become reliant on the gender of the WM as being the correct response during the visual search array. No such pattern emerged from a visual inspection of RT plots for each participant indicated stable patterns throughout the task. Moreover, a bypass of the visual search component would imply no relationship between the emotional valence of the WM representation and that of the target in the visual search display. However, we found an effect of congruency; faster RTs to targets in the visual search array matching the emotional valence of the WM representation. This demonstrated identification of target and sufficient engagement to detect congruency.

Whilst there is evidence of engagement with both components of the task, a second issue to consider is whether participants engaged with the search aspect of the visual search task. The visual search and associated gender identification task were in line with previous work [43]. The contrast and luminance histograms of the pictures were equalized, minimising feature-based search; instead, attention was drawn to the targets using colour singletons (i.e., bottom-up salience; [83]). Consequently, if the engagement with the visual search array observed in the data was not due to top-down modulation of visual search (i.e., search of the target and identification of gender, as proposed), it may be due to bottom-up salience of the colour singleton. Whilst this seems unlikely, as this pattern of attention allocation would not explain our findings, it would still be indicative of engagement in visual search by identifying a target among distractors.

Finally, if participants had engaged in a confirmatory form of visual search whereby they attended to the target merely to confirm that it corresponds to their WM template: a) a learning pattern as indicated by faster RTs would be expected, which is not present in the data and b) this would require a search for the target as indicated by the colour singleton and comparison to the representation held in WM. If this were the case, targets corresponding to WM representation would strengthen (or reinforce) the representation [84] meaning participants would find it easier to identify a matching representation in a match/no-match test. However, we did not find an effect of congruency on percentage of correct responses, suggesting participants did not engage in such a form of visual search. Future research should further explore these issues by varying identity and emotional valences of WM templates and targets.

## Conclusion

In summary, we found evidence of a WM modulated attentional bias to naturalistic emotional faces. Higher antisocial traits predicted an increased WM bias of visual perception, meaning that participants displaying higher aggression and trait anger were more efficient in identifying emotional faces identical to the induced internal representations. Finally, we found a relationship between the WM modulated bias of visual perception and later stages in social information processing including emotional response, hostile attribution bias, and response enactment variables. The present research demonstrated the role of emotional WM templates in social information processing, from encoding social stimuli to behavioural outcomes. Moreover, these findings suggested that WM biases of perception may contribute to the development and maintenance of cognitive biases related to antisocial traits.

## Supporting information

S1 Table Correlation matrix for trait anger, aggression, and scores on the SEIP-Q. (DOCX)

S2 Table Means and standard deviations for aggression, trait anger, attributional and emotional response variables. (DOCX)

S1 File Further detail on the development and validation of the dual task. (DOCX)

S2 File The extended pre-registered exploration of links between WM modulations of visual attention and patterns of social information processing (Tables 7, 8, and 9). (DOCX)

## References

[1] Assink M, van der Put CE, Hoeve M, de Vries SL, Stams GJ, Oort FJ. Risk factors for persistent delinquent behavior among juveniles: A meta-analytic review. Clinical Psychology Review. 2015 Dec 1;42:47–61. doi: 10.1016/j.cpr.2015.08.002 26301752

[2] Cohen MA, Piquero AR, Jennings WG. Studying the costs of crime across offender trajectories. Criminology & Public Policy. 2010 May;9(2):279–305. doi: 10.1111/j.1745-9133.2010.00627.x

[3] Boxer P, Dubow EF. A social-cognitive information-processing model for school-based aggression reduction and prevention programs: Issues for research and practice. Applied and Preventive Psychology. 2001 Jun 1;10(3):177–92. doi: 10.1016/S0962-1849(01)80013-5

[4] Vaske J, Galyean K, Cullen FT. Toward a biosocial theory of offender rehabilitation: Why does cognitive-behavioral therapy work?. Journal of Criminal Justice. 2011 Jan 1;39(1):90–102. doi: 10.1016/j.jcrimjus.2010.12.006

[5] Cornet LJ, van der Laan PH, Nijman HL, Tollenaar N, de Kogel CH. Neurobiological factors as predictors of prisoners’ response to a cognitive skills training. Journal of Criminal Justice. 2015 Mar 1;43(2):122–32. doi: 10.1016/j.jcrimjus.2015.02.003

[6] Haselton MG, Nettle D, Murray DR. The evolution of cognitive bias. The handbook of evolutionary psychology. 2015 Nov 23:1–20. doi: 10.1002/9780470939376.ch25

[7] Chabrol H, Goutaudier N, Melioli T, van Leeuwen N, Gibbs JC. Impact of antisocial behavior on psychopathic traits in a community sample of adolescents. Bulletin of the Menninger Clinic. 2014 Sep;78(3):228–42. doi: 10.1521/bumc.2014.78.3.228 25247742

[8] Crick NR, Dodge KA. A review and reformulation of SIP mechanisms in children social adjustment. Psychological Bulletin. 1994 115(1): 74–101.

[9] Dodge KA, Godwin J. Conduct Problems Prevention Research Group Social-information-processing patterns mediate the impact of preventive intervention on adolescent antisocial behavior. Psychological Science. 2013 Apr;24(4):456–65. doi: 10.1177/0956797612457394 23406610 PMC3726052

[10] Fontaine RG, Dodge KA. Real-time decision making and aggressive behavior in youth: A heuristic model of response evaluation and decision (RED). Aggressive Behavior: Official Journal of the International Society for Research on Aggression. 2006 Nov 1;32(6):604–24. doi: 10.1002/ab.20150 20802851 PMC2928648

[11] Dodge KA, Pettit GS, Bates JE, Valente E. Social information-processing patterns partially mediate the effect of early physical abuse on later conduct problems. Journal of Abnormal Psychology. 1995 Nov;104(4):632. doi: 10.1037//0021-843x.104.4.632 8530766

[12] Smeijers D, Bulten EB, Brazil IA. The Computations of hostile biases (CHB) model: Grounding hostility biases in a unified cognitive framework. Clinical psychology review. 2019 Nov 1;73:101775. doi: 10.1016/j.cpr.2019.101775 31726277

[13] Manning KE. Seeing red? A systematic review of the evidence for attentional biases to threat-relevant stimuli in propensity to reactive aggression. Aggression and violent behavior. 2020 Jan 1(50):101359. doi: 10.1016/j.avb.2019.101359

[14] Repple J, Pawliczek CM, Voss B, Siegel S, Schneider F, Kohn N, et al. From provocation to aggression: the neural network. BMC neuroscience. 2017 Dec;18(1):1–9. doi: 10.1186/s12868-016-0326-z 29041906 PMC5646154

[15] Coccaro EF, Fanning J, Lee R. Development of a social emotional information processing assessment for adults (SEIP-Q). Aggressive behavior. 2017 Jan;43(1):47–59. doi: 10.1002/ab.21661 27321909 PMC6323644

[16] Elbert T, Moran JK, Schauer M. Lust for violence: appetitive aggression as a fundamental part of human nature. e- Neuroforum. 2017;23(2):77–84.

[17] Wrangham RW. Two types of aggression in human evolution. Proceedings of the National Academy of Sciences. 2018 Jan 9;115(2):245–53. doi: 10.1073/pnas.1713611115 29279379 PMC5777045

[18] Lobbestael J, Cousijn J, Brugman S, Wiers RW. Approach and avoidance towards aggressive stimuli and its relation to reactive and proactive aggression. Psychiatry Research. 2016 Jun 30;240:196–201. doi: 10.1016/j.psychres.2016.04.038 27111213

[19] Maoz K, Adler AB, Bliese PD, Sipos ML, Quartana PJ, Bar-Haim Y. Attention and interpretation processes and trait anger experience, expression, and control. Cognition and Emotion. 2017 Oct 3;31(7):1453–64. doi: 10.1080/02699931.2016.1231663 27653208

[20] Blair RJ, Mitchell DG. Psychopathy, attention and emotion. Psychological Medicine. 2009 Apr;39(4):543. doi: 10.1017/S0033291708003991 18700991 PMC2769016

[21] Li J, Fraser MW, Wike TL. Promoting social competence and preventing childhood aggression: A framework for applying social information processing theory in intervention research. Aggression and Violent Behavior. 2013 May 1;18(3):357–64. doi: 10.1016/j.avb.2013.01.001

[22] Yao N, Rodriguez MA, He M, Qian M. The influence of visual working memory representations on attention bias to threat in individuals with high trait anxiety. Journal of Experimental Psychopathology. 2019 Oct 11;10(4):1–15. doi: 10.1177/2043808719876149

[23] Battistoni E, Stein T, Peelen MV. Preparatory attention in visual cortex. Annals of the New York Academy of Sciences. 2017 May;1396(1):92–107. doi: 10.1111/nyas.13320 28253445

[24] Soto D, Humphreys GW. Stressing the mind: The effect of cognitive load and articulatory suppression on attentional guidance from working memory. Perception & Psychophysics. 2008 Jul;70(5):924–34. doi: 10.3758/pp.70.5.924 18613638

[25] Carlisle NB. Focus: Attention Science: Flexibility in Attentional Control: Multiple Sources and Suppression. The Yale Journal of Biology and Medicine. 2019 Mar;92(1):103–113. 30923477 PMC6430179

[26] Salahub C, Emrich SM. Fear not! Anxiety biases attentional enhancement of threat without impairing working memory filtering. Cognitive, Affective, & Behavioral Neuroscience. 2020 Dec;20(6):1248–60. doi: 10.3758/s13415-020-00831-3 32948915

[27] van Moorselaar D, Theeuwes J, Olivers CN. In competition for the attentional template: Can multiple items within visual working memory guide attention?. Journal of Experimental Psychology: Human Perception and Performance. 2014 Aug;40(4):1450–1465. doi: 10.1037/a0036229 24730738

[28] Cisler JM, Koster EH. Mechanisms of attentional biases towards threat in anxiety disorders: An integrative review. Clinical Psychology Review. 2010 Mar 1;30(2):203–216. doi: 10.1016/j.cpr.2009.11.003 20005616 PMC2814889

[29] Day A, Daffern M, Polaschek D, Dunne A, Senn A. The classification of people with a history of interpersonal violence for correctional treatment purposes: Possibilities for a schema-informed approach. Aggression and Violent Behavior. 2020 Jun 15;101450. doi: 10.1016/j.avb.2020.101450

[30] Gilbert F, Daffern M, Talevski D, Ogloff JR. The role of aggression-related cognition in the aggressive behavior of offenders: A general aggression model perspective. Criminal Justice and Behavior. 2013 Feb;40(2):119–38. doi: 10.1177/0093854812467943

[31] Ogilvie JM, Stewart AL, Chan RC, Shum DH. Neuropsychological measures of executive function and antisocial behavior: A meta-analysis. Criminology. 2011 Nov;49(4):1063–107. doi: 10.1111/j.1745-9125.2011.00252.x

[32] Kumar S, Soto D, Humphreys GW. Electrophysiological evidence for attentional guidance by the contents of working memory. European Journal of Neuroscience. 2009 Jul;30(2):307–317. doi: 10.1111/j.1460-9568.2009.06805.x 19691812

[33] Carlisle NB, Woodman GF. Reconciling conflicting electrophysiological findings on the guidance of attention by working memory. Attention, Perception, & Psychophysics. 2013 Oct;75(7):1330–1335. doi: 10.3758/s13414-013-0529-7 23918552 PMC3800228

[34] Burra N, Kerzel D. Task demands modulate effects of threatening faces on early perceptual encoding. Frontiers in psychology. 2019 Oct 24;10:2400. doi: 10.3389/fpsyg.2019.02400 31708839 PMC6821787

[35] Jackson MC, Linden DE, Raymond JE. Angry expressions strengthen the encoding and maintenance of face identity representations in visual working memory. Cognition & Emotion. 2014 Feb 17;28(2):278–297. doi: 10.1080/02699931.2013.816655 23895082

[36] Van Nieuwenhuijzen M, Van Rest MM, Embregts PJ, Vriens A, Oostermeijer S, Van Bokhoven I, et al. Executive functions and social information processing in adolescents with severe behavior problems. Child Neuropsychology. 2017 Feb 17;23(2):228–41. doi: 10.1080/09297049.2015.1108396 26563817

[37] Cohen J. Statistical power analysis for the behavioral sciences. 2 nd ed. New York: Academic press; 1969.

[38] Raine A, Dodge K, Loeber R, Gatzke-Kopp L, Lynam D, Reynolds C, et al. The reactive–proactive aggression questionnaire: Differential correlates of reactive and proactive aggression in adolescent boys. Aggressive Behavior: Official Journal of the International Society for Research on Aggression. 2006 Apr;32(2):159–171. doi: 10.1002/ab.20115 20798781 PMC2927832

[39] Cenkseven-Önder F, Avci R, Çolakkadioglu O. Validity and Reliability of the Reactive-Proactive Aggression Questionnaire in Turkish Adolescents. Educational Research and Reviews. 2016 Oct 23;11(20):1931–1943. doi: 10.5897/ERR2016.2937

[40] Spielberger C D. State-Trait Anger Expression Inventory-2. Lutz: Psychological Assessment Resources; 1999.

[41] Wang X, Yang L, Yang J, Gao L, Zhao F, Xie X, et al. Trait anger and aggression: A moderated mediation model of anger rumination and moral disengagement. Personality and Individual Differences. 2018 Apr 15;125:44–49. doi: 10.1016/j.paid.2017.12.029

[42] Lievaart M, Franken IH, Hovens JE. Anger assessment in clinical and nonclinical populations: Further validation of the State–Trait Anger Expression Inventory‐2. Journal of Clinical Psychology. 2016 Mar;72(3):263–278. doi: 10.1002/jclp.22253 26766132

[43] Burra N, Coll SY, Barras C, Kerzel D. Electrophysiological evidence for attentional capture by irrelevant angry facial expressions: Naturalistic faces. Neuroscience Letters. 2017 Jan 10;637:44–49. doi: 10.1016/j.neulet.2016.11.055 27899310

[44] Tottenham N, Tanaka JW, Leon AC, McCarry T, Nurse M, Hare TA, et al. The NimStim set of facial expressions: judgments from untrained research participants. Psychiatry Research. 2009 Aug 15;168(3):242–249. doi: 10.1016/j.psychres.2008.05.006 19564050 PMC3474329

[45] Willenbockel V, Sadr J, Fiset D, Horne GO, Gosselin F, Tanaka JW. Controlling low-level image properties: the SHINE toolbox. Behavior Research Methods. 2010 Aug;42(3):671–684. doi: 10.3758/BRM.42.3.671 20805589

[46] RStudio Team (2020). RStudio: Integrated Development for R [Internet]. Boston: RStudio, PBC; 2020 [updated 2020; cited 2021 Apr 3]. http://www.rstudio.com/.

[47] Price RB, Kuckertz JM, Siegle GJ, Ladouceur CD, Silk JS, Ryan ND, et al. Empirical recommendations for improving the stability of the dot-probe task in clinical research. Psychological assessment. 2015 Jun;27(2):365–376. doi: 10.1037/pas0000036 25419646 PMC4442069

[48] Soto D, Greene CM, Kiyonaga A, Rosenthal CR, Egner T. A parieto-medial temporal pathway for the strategic control over working memory biases in human visual attention. Journal of Neuroscience. 2012 Dec 5;32(49):17563–17571. doi: 10.1523/JNEUROSCI.2647-12.2012 23223280 PMC6621679

[49] Zvielli A, Bernstein A, Koster EH. Temporal dynamics of attentional bias. Clinical Psychological Science. 2015 Sep;3(5):772–788. doi: 10.1177/2167702614551572

[50] Mazza V, Dallabona M, Chelazzi L, Turatto M. Cooperative and opposing effects of strategic and involuntary attention. Journal of Cognitive Neuroscience. 2011 Oct;23(10):2838–2851. doi: 10.1162/jocn.2011.21634 21265602

[51] Krusemark EA, Kiehl KA, Newman JP. Endogenous attention modulates early selective attention in psychopathy: An ERP investigation. Cognitive, Affective, & Behavioral Neuroscience. 2016 Oct;16(5):779–788. doi: 10.3758/s13415-016-0430-7 27225501 PMC6135064

[52] Pan Y, Soto D. The modulation of perceptual selection by working memory is dependent on the focus of spatial attention. Vision Research. 2010 Jul 9;50(15):1437–1444. doi: 10.1016/j.visres.2009.10.016 19883679

[53] Berggren N, Eimer M. Object-based target templates guide attention during visual search. Journal of Experimental Psychology: Human Perception and Performance. 2018 Sep;44(9):1368–1382. doi: 10.1037/xhp0000541 29723006

[54] Moriya J, Koster EH, De Raedt R. The influence of working memory on the anger superiority effect. Cognition and Emotion. 2014 Nov 17;28(8):1449–64. doi: 10.1080/02699931.2014.890094 24564850

[55] Fan L, Ding C, Guo R, Xu M, Diao L, Yang D. Visual working memory representations guide the detection of emotional faces: An ERP study. Vision Research. 2016 Feb 1;119:1–8. doi: 10.1016/j.visres.2015.08.021 26731647

[56] Bar-Haim Y, Lamy D, Pergamin L, Bakermans-Kranenburg MJ, Van Ijzendoorn MH. Threat-related attentional bias in anxious and nonanxious individuals: a meta-analytic study. Psychological Bulletin. 2007 Jan;133(1):1–24. doi: 10.1037/0033-2909.133.1.1 17201568

[57] Wieser MJ, Hambach A, Weymar M. Neurophysiological correlates of attentional bias for emotional faces in socially anxious individuals–Evidence from a visual search task and N2pc. Biological Psychology. 2018 Feb 1;132:192–201. doi: 10.1016/j.biopsycho.2018.01.004 29307540

[58] Taylor AJ, Jose M. Physical aggression and facial expression identification. Europe’s Journal of Psychology. 2014 Nov 28;10(4):650–659. doi: 10.5964/ejop.v10i4.816

[59] Crago RV, Renoult L, Biggart L, Nobes G, Satmarean T, Bowler JO. Physical aggression and attentional bias to angry faces: an event related potential study. Brain Research. 2019 Nov 15;1723:146387. doi: 10.1016/j.brainres.2019.146387 31419430

[60] Oostermeijer S, Nieuwenhuijzen M, Van de Ven PM, Popma A, Jansen LM. Social information processing problems related to reactive and proactive aggression of adolescents in residential treatment. Personality and Individual Differences. 2016 Feb 1;90:54–60. doi: 10.1016/j.paid.2015.10.035

[61] Dodge KA. Translational science in action: Hostile attributional style and the development of aggressive behavior problems. Development and Psychopathology. 2006;18(3):791–814. doi: 10.1017/s0954579406060391 17152401 PMC2745254

[62] Soto D, Humphreys GW. Automatic guidance of visual attention from verbal working memory. Journal of Experimental Psychology: Human Perception and Performance. 2007 Jun;33(3):730–757. doi: 10.1037/0096-1523.33.3.730 17563233

[63] Maran T, Sachse P, Furtner M. From specificity to sensitivity: affective states modulate visual working memory for emotional expressive faces. Frontiers in Psychology. 2015 Aug 27;6:1297. doi: 10.3389/fpsyg.2015.01297 26379609 PMC4550750

[64] Jackson MC, Wu CY, Linden DE, Raymond JE. Enhanced visual short-term memory for angry faces. Journal of Experimental Psychology: Human Perception and Performance. 2009 Apr;35(2):363–374. doi: 10.1037/a0013895 19331494

[65] Bays PM, Catalao RF, Husain M. The precision of visual working memory is set by allocation of a shared resource. Journal of Vision. 2009 Sep 1;9(10):7, 1–11. doi: 10.1167/9.10.7 19810788 PMC3118422

[66] Öhman A, Mineka S. Fears, phobias, and preparedness: toward an evolved module of fear and fear learning. Psychological Review. 2001 Jul;108(3):483–522. doi: 10.1037/0033-295x.108.3.483 11488376

[67] Soto D, Heinke D, Humphreys GW, Blanco MJ. Early, involuntary top-down guidance of attention from working memory. Journal of Experimental Psychology: Human Perception and Performance. 2005 Apr;31(2):248–261. doi: 10.1037/0096-1523.31.2.248 15826228

[68] Yin S, Bi T, Chen A, Egner T. Ventromedial prefrontal cortex drives the prioritization of self-associated stimuli in working memory. Journal of Neuroscience. 2021 Mar 3;41(9):2012–23. doi: 10.1523/JNEUROSCI.1783-20.2020 33462089 PMC7939096

[69] Miller NV, Johnston C. Social threat attentional bias in childhood: Relations to aggression and hostile intent attributions. Aggressive behavior. 2019 May;45(3):245–54. doi: 10.1002/ab.21813 30635910

[70] Bushman BJ. Violent media and hostile appraisals: A meta-analytic review. Aggressive behavior. 2016 Nov;42(6):605–13. doi: 10.1002/ab.21655 27121494

[71] Mathys C, Daunizeau J, Friston KJ, Stephan KE. A Bayesian foundation for individual learning under uncertainty. Frontiers in human neuroscience. 2011 May 2;5:39. doi: 10.3389/fnhum.2011.00039 21629826 PMC3096853

[72] Friston K. The free-energy principle: a unified brain theory?. Nature reviews neuroscience. 2010 Feb;11(2):127–38. doi: 10.1038/nrn2787 20068583

[73] Gerin W, Davidson KW, Christenfeld NJ, Goyal T, Schwartz JE. The role of angry rumination and distraction in blood pressure recovery from emotional arousal. Psychosomatic medicine. 2006 Jan 1;68(1):64–72. doi: 10.1097/01.psy.0000195747.12404.aa 16449413

[74] Wilkowski BM, Robinson MD. The anatomy of anger: An integrative cognitive model of trait anger and reactive aggression. Journal of personality. 2010 Feb;78(1):9–38. doi: 10.1111/j.1467-6494.2009.00607.x 20433611

[75] Wilkowski BM, Robinson MD, Meier BP. Agreeableness and the prolonged spatial processing of antisocial and prosocial information. Journal of Research in Personality. 2006 Dec 1;40(6):1152–68.

[76] Wilkowski BM, Robinson MD, Gordon RD, Troop-Gordon W. Tracking the evil eye: Trait anger and selective attention within ambiguously hostile scenes. Journal of Research in Personality. 2007 Jun 1;41(3):650–66. doi: 10.1016/j.jrp.2006.07.003 24920865 PMC4049355

[77] Buades-Rotger M, Krämer UM. From words to action: Implicit attention to antisocial semantic cues predicts aggression and amygdala reactivity to angry faces in healthy young women. Aggressive behavior. 2018 Nov;44(6):624–37. doi: 10.1002/ab.21787 30141188

[78] Coelho CM, Cloete S, Wallis G. The face-in-the-crowd effect: When angry faces are just cross (es). Journal of Vision. 2010 Jan 2;10(1):7, 1–14. doi: 10.1167/10.1.7 20143900

[79] Burra N, Barras C, Coll SY, Kerzel D. Electrophysiological evidence for attentional capture by irrelevant angry facial expressions. Biological Psychology. 2016 Oct 1;120:69–80. doi: 10.1016/j.biopsycho.2016.08.008 27568328

[80] Armitage J, Eerola T. Reaction time data in music cognition: Comparison of pilot data from lab, crowdsourced, and convenience Web samples. Frontiers in Psychology. 2020 Jan 8;10:2883. doi: 10.3389/fpsyg.2019.02883 31969849 PMC6960264

[81] Miloff A, Savva A, Carlbring P. Cognitive bias measurement and social anxiety disorder: correlating self-report data and attentional bias. Internet Interventions. 2015 Sep 1;2(3):227–234. doi: 10.1016/j.invent.2015.03.006

[82] Miller JD, Crowe M, Weiss B, Maples-Keller JL, Lynam DR. Using online, crowdsourcing platforms for data collection in personality disorder research: The example of Amazon’s Mechanical Turk. Personality Disorders: Theory, Research, and Treatment. 2017 Jan;8(1):26–34. doi: 10.1037/per0000191 28045305

[83] Wolfe JM, Horowitz TS. Five factors that guide attention in visual search. Nature Human Behaviour. 2017 Mar 8;1(3):1–8. doi: 10.1038/s41562-017-0058 PMC9879335 36711068

[84] Souza AS, Oberauer K. In search of the focus of attention in working memory: 13 years of the retro-cue effect. Attention, Perception, & Psychophysics. 2016 Oct;78(7):1839–60. doi: 10.3758/s13414-016-1108-5 27098647

