# Supplementary material for: Working memory guidance of visual attention to threat in offenders
Source: PLoS One. 2022 Jan 7;17(1):e0261882. doi: 10.1371/journal.pone.0261882 (PMC8741051; doi:10.1371/journal.pone.0261882)
Supplement: S1 Table — (DOCX) [file pone.0261882.s001.docx]

**Table 5. Correlation matrix for Trait Anger, Aggression, and scores on the SEIP-Q.**

|  |  | 1 | 2 | 3 | 4 | 5 | 6 | 7 | 8 | 9 | 10 | 11 | 12 |
| --- | --- | --- | --- | --- | --- | --- | --- | --- | --- | --- | --- | --- | --- |
| 1 | BS1-away parameter | *-* |  |  |  |  |  |  |  |  |  |  |  |
| 2 | BS1- toward parameter | *-.58**** | *-* |  |  |  |  |  |  |  |  |  |  |
| 3 | BS3- away parameter | *.78**** | *-.55**** | *-* |  |  |  |  |  |  |  |  |  |
| 4 | Trait Anger | *-.25*** | *.25*** | *-.14* | *-* |  |  |  |  |  |  |  |  |
| 5 | Reactive Aggression | *-.16* | *.25*** | *-.08* | *.63**** | *-* |  |  |  |  |  |  |  |
| 6 | Hostile Attribution | *-.21** | *.20** | *-.24*** | *.27*** | *.16* | *-* |  |  |  |  |  |  |
| 7 | Instrumental Attribution | *-.03* | *.11* | *.03* | *-.16* | *-.21** | *.08* | *-* |  |  |  |  |  |
| 8 | Benign Attribution | *.16* | *-.12* | *.06* | *-.15* | *-.17* | *-.43**** | *-.06* | *-* |  |  |  |  |
| 9 | Negative Emotional Response | *.04* | *.07* | *.13* | *.12* | *.13* | *.37**** | *.25*** | *-.19** | *-* |  |  |  |
| 10 | Response Enactment: Appropriate | *-.04* | *.06* | *-.09* | *.04* | *.03* | *.13* | *.14* | *.33**** | *.06* | *-* |  |  |
| 11 | Response Enactment: Direct Aggression | *-.18* | *.17* | *-.21** | *.55**** | *.51**** | *.54**** | *-.17* | *-.27*** | *.30*** | *-.01* | *-* |  |
| 12 | Response Enactment: Relational Aggression | *-.27*** | *.29*** | *-.12* | *.37**** | *.35**** | *.56**** | *.05* | *-.36**** | *.49**** | *-.06* | *.63**** | *-* |

**p<0.05, **p<0.01, ***p<0.001.*
