# Supplementary material for: Working memory guidance of visual attention to threat in offenders
Source: PLoS One. 2022 Jan 7;17(1):e0261882. doi: 10.1371/journal.pone.0261882 (PMC8741051; doi:10.1371/journal.pone.0261882)
Supplement: S2 Table — (DOCX) [file pone.0261882.s002.docx]

**Table 6. Means and Standard Deviations for Aggression, Trait Anger, Attributional and Emotional response variables.**

| *Measure* | *Mean* | *Standard Deviation* |
| --- | --- | --- |
| Reactive Proactive Questionnaire | 11.67 | 6 |
| Reactive Aggression scores | 8.84 | 3.75 |
| Trait Anger | 20.55 | 5.19 |
| Hostile attribution | 2.1 | 0.4 |
| Instrumental attribution | 3.3 | 0.3 |
| Negative evaluation and response | 2.8 | 0.4 |
| Benign attribution | 2.55 | 0.35 |
