# Supplementary material for: Working memory guidance of visual attention to threat in offenders
Source: PLoS One. 2022 Jan 7;17(1):e0261882. doi: 10.1371/journal.pone.0261882 (PMC8741051; doi:10.1371/journal.pone.0261882)
Supplement: S1 File — (DOCX) [file pone.0261882.s003.docx]

Dual Task- Development and validation

The development of the dual task was informed by the biased competition model of visual selection [1] according to which neural activity is biased in favour of object features held in WM. Facilitation of visual attention by WM is typically studied by asking participants to memorise content (a WM template) overlapping with the target of attention [2]. Guidance of attention by WM has been observed at early stages during encoding [3] as well as during and after information is consolidated in WM [4]. Moreover, the guidance was absent when cues were primed but not encoded in WM for later report [3]. This effect has been replicated in studies using functional magnetic resonance imaging [5] and event-related potentials [6]. The paradigm has been applied to visual search displays containing shapes and simple objects [3, 2], and more recently to real-world objects and scenes [7] and facial expressions [8].

In line with previous research, participants in this study were required to complete the (dual) combined WM and visual search task. Only one face was displayed during encoding of the WM template due to limited capacity for face memory [9, 10]. To ensure that the encoding of the WM template was directly relevant to the task [11] the match/no-match test was displayed at the end of each trial. The search array following the encoding display was a similar design to the visual search task using naturalistic face stimuli developed by Burra et al [12]. As WM templates and targets had the same identity (and the same emotional valence in half of the trials), additional checks were conducted to exclude the possibility of participants responding to the gender of the target based on the memorised WM template. Specifically, RTs were plotted and inspected for each participant and revealed a stable RT pattern as opposed to a learning pattern, indicative of reliance upon information stored in WM.

**References**

1. Desimone R, Duncan J. Neural mechanisms of selective visual attention. Annual review of neuroscience. 1995 Mar;18(1):193-222.

2. Downing PE. Interactions between visual working memory and selective attention. Psychol Sci. 2000;11(6):467–73.

3. Soto D, Heinke D, Humphreys GW, Blanco MJ. Early, involuntary top-down guidance of attention from working memory. Journal of Experimental Psychology: Human Perception and Performance. 2005 Apr;31(2):248- 261. doi: ﻿10.1037/0096-1523.31.2.248.

4. Olivers CN, Meijer F, Theeuwes J. Feature-based memory-driven attentional capture: visual working memory content affects visual attention. Journal of Experimental Psychology: Human Perception and Performance. 2006 Oct;32(5):1243- 1265.

5. Soto D, Greene CM, Kiyonaga A, Rosenthal CR, Egner T. A parieto-medial temporal pathway for the strategic control over working memory biases in human visual attention. Journal of Neuroscience. 2012 Dec 5;32(49):17563-17571. doi: ﻿10.1523/JNEUROSCI.2647-12.2012.

6. Kumar S, Soto D, Humphreys GW. Electrophysiological evidence for attentional guidance by the contents of working memory. European Journal of Neuroscience. 2009 Jul;30(2):307-317. doi: ﻿10.1111/j.1460-9568.2009.06805.x.

7. Seidl-Rathkopf KN, Turk-Browne NB, Kastner S. Automatic guidance of attention during real-world visual search. Attention, Perception, & Psychophysics. 2015 Aug;77(6):1881-95. doi: ﻿10.3758/s13414-015-0903-8.

8. Yao N, Rodriguez MA, He M, Qian M. The influence of visual working memory representations on attention bias to threat in individuals with high trait anxiety. Journal of Experimental Psychopathology. 2019 Oct 11;10(4):1-15. doi: ﻿10.1177/2043808719876149.

9. Towler J, Kelly M, Eimer M. The focus of spatial attention determines the number and precision of face representations in working memory. Cerebral Cortex. 2016 Jun 1;26(6):2530-40. doi: ﻿10.1093/cercor/bhv083.

10. van Moorselaar D, Theeuwes J, Olivers CN. In competition for the attentional template: Can multiple items within visual working memory guide attention?. Journal of Experimental Psychology: Human Perception and Performance. 2014 Aug;40(4):1450-1465. doi: ﻿10.1037/a0036229.

11. Woodman GF, Carlisle NB, Reinhart RM. Where do we store the memory representations that guide attention?. Journal of vision. 2013 Sep 1;13(3):1-17. doi: 10.1167/13.3.1.

12. Burra N, Coll SY, Barras C, Kerzel D. Electrophysiological evidence for attentional capture by irrelevant angry facial expressions: Naturalistic faces. Neuroscience Letters. 2017 Jan 10;637:44-49. doi: ﻿10.1016/j.neulet.2016.11.055.
