# Supplementary material for: Working memory guidance of visual attention to threat in offenders
Source: PLoS One. 2022 Jan 7;17(1):e0261882. doi: 10.1371/journal.pone.0261882 (PMC8741051; doi:10.1371/journal.pone.0261882)
Supplement: S2 File — (DOCX) [file pone.0261882.s004.docx]

## **Exploratory Analyses**

## **Are WM visual selection biases predicted by social-emotional information processing variables?**

The third hypothesis investigated the relationship between the effect of WM on visual selection bias and later stages in social information processing. Higher hostile attribution scores were expected to predict attentional bias toward angry faces when holding an angry face in WM compared to neutral faces when holding a neutral face in WM (BS1_POSITIVE_) and slower RTs to (or attentional bias away from) neutral faces when holding an angry face in WM compared to neutral faces when holding a neutral face in WM (BS3) arrays. Hierarchical regressions were planned to evaluate the relationship between attentional bias (BS1 and BS3 as DVs) and later stages in SIP (as predictors). Age, gender, and education were added at step 1, hostile attribution scores were added at step 2, instrumental attribution and negative emotional response scores were added at step 3, and response enactment (for aggressive responses) scores were added at step 4.

Results

Response enactment for aggressive responses was a positive predictor of attentional bias toward angry faces whilst holding an angry face in mind (BS1_POSITIVE_, β = 0.28, p= 0.04, model at step 4) and a negative predictor of attentional bias away from angry faces whilst holding an angry face in mind (BS1_NEGATIVE_, β = -0.27, p= 0.04, model at step 4). Negative emotional response was a significant positive predictor of attentional bias away from angry faces whilst holding an angry face in mind (BS1_NEGATIVE_, β = 0.23, p= 0.04, model at step 4) and of attentional bias away from neutral faces identified whilst holding an angry face in mind (BS2_NEGATIVE_, β = 0.27, p= 0.01, model at step 3). Hostile attribution scores were also a significant negative predictor of attentional bias away from neutral faces identified whilst holding an angry face in mind (BS2_NEGATIVE_, β= -0.34, p= 0.001, model at step 3). A summary of hierarchical regression models is shown in Tables 7-9.

**Table 7.** **Hierarchical multiple linear regression analysis predicting attentional bias toward angry faces whilst holding an angry face in WM (BS1_POSITIVE_) with SEIP-Q variables as predictors.**

|  | ***β*** | ***SE*** | ***p*** | ***R^2^ _Total/Change_*** |
| --- | --- | --- | --- | --- |
| *Step 1* |  |  |  | *0.003* |
| Gender | -0.08 | 0.10 | 0.41 |  |
| Education | 0.06 | 0.10 | 0.53 |  |
| Age | -0.14 | 0.10 | 0.15 |  |
| *Step 2* |  |  |  | *0.02/ 0.07* |
| Gender | -0.06 | 0.09 | 0.51 |  |
| Education | 0.05 | 0.10 | 0.63 |  |
| Age | -0.13 | 0.10 | 0.20 |  |
| Hostile Attribution | 0.17 | 0.09 | 0.08 |  |
| *Step 3* |  |  |  | *0.02/0.53* |
| Gender | -0.07 | 0.10 | 0.48 |  |
| Education | 0.03 | 0.10 | 0.77 |  |
| Age | -0.13 | 0.09 | 0.18 |  |
| Hostile Attribution | 0.17 | 0.10 | 0.10 |  |
| Instrumental Attribution | 0.11 | 0.10 | 0.27 |  |
| Negative Emotional Response | -0.03 | 0.11 | 0.77 |  |
| *Step 4* |  |  |  | *0.05/0.04** |
| Gender | -0.03 | 0.10 | 0.75 |  |
| Education | 0.05 | 0.10 | 0.58 |  |
| Age | -0.08 | 0.10 | 0.40 |  |
| Hostile Attribution | 0.03 | 0.12 | 0.80 |  |
| Instrumental Attribution | 0.15 | 0.10 | 0.13 |  |
| Negative Emotional Response | -0.11 | 0.11 | 0.32 |  |
| Response Enactment (aggressive responses) | 0.28 | 0.13 | 0.04* |  |

*β*, standardized regression coefficient.

**p* < .05.

**Table 8.** **Hierarchical multiple linear regression analysis predicting attentional bias away from angry faces whilst holding an angry face in WM (BS1_NEGATIVE_) with SEIP-Q variables as predictors.**

|  | ***β*** | ***SE*** | ***p*** | ***R^2^ _Total/Change_*** |
| --- | --- | --- | --- | --- |
| *Step 1* |  |  |  | *0.03* |
| Gender | 0.15 | 0.09 | 0.10 |  |
| Education | -0.02 | 0.09 | 0.84 |  |
| Age | 0.19 | 0.10 | 0.05 |  |
| *Step 2* |  |  |  | *0.05/0.07* |
| Gender | 0.14 | 0.09 | 0.14 |  |
| Education | -0.004 | 0.09 | 0.96 |  |
| Age | 0.17 | 0.09 | 0.08 |  |
| Hostile Attribution | -0.17 | 0.09 | 0.07 |  |
| *Step 3* |  |  |  | *0.05/0.32* |
| Gender | 0.11 | 0.10 | 0.26 |  |
| Education | 0.03 | 0.10 | 0.79 |  |
| Age | 0.18 | 0.09 | 0.06 |  |
| Hostile Attribution | -0.23 | 0.10 | 0.03 |  |
| Instrumental Attribution | -0.07 | 0.10 | 0.50 |  |
| Negative Emotional Response | 0.16 | 0.11 | 0.15 |  |
| *Step 4* |  |  |  | *0.08/0.04** |
| Gender | 0.07 | 0.10 | 0.46 |  |
| Education | 0.001 | 0.10 | 0.99 |  |
| Age | 0.14 | 0.10 | 0.15 |  |
| Hostile Attribution | -0.09 | 0.12 | 0.44 |  |
| Instrumental Attribution | -0.11 | 0.10 | 0.27 |  |
| Negative Emotional Response | 0.23 | 0.11 | 0.04* |  |
| Response Enactment (aggressive responses) | -0.27 | 0.13 | 0.04* |  |

*β*, standardized regression coefficient.

**p* < .05.

**Table 9. ﻿ Hierarchical multiple linear regression analysis predicting attentional bias away from neutral faces whilst holding an angry face in WM (BS2_NEGATIVE_) with SEIP-Q variables as predictors.**

|  | ***β*** | ***SE*** | ***p*** | ***R^2^ _Total/Change_*** |
| --- | --- | --- | --- | --- |
| *Step 1* |  |  |  | *-0.01* |
| Gender | 0.06 | 0.10 | 0.55 |  |
| Education | -0.07 | 0.10 | 0.50 |  |
| Age | 0.05 | 0.10 | 0.64 |  |
| *Step 2* |  |  |  | *0.03/0.01** |
| Gender | 0.03 | 0.09 | 0.70 |  |
| Education | -0.04 | 0.09 | 0.63 |  |
| Age | 0.02 | 0.09 | 0.80 |  |
| Hostile Attribution | -0.23 | 0.09 | 0.02* |  |
| *Step 3* |  |  |  | *0.07/0.04** |
| Gender | -0.03 | 0.10 | 0.82 |  |
| Education | -0.01 | 0.10 | 0.94 |  |
| Age | 0.04 | 0.09 | 0.64 |  |
| Hostile Attribution | -0.34 | 0.12 | 0.001** |  |
| Instrumental Attribution | -0.01 | 0.10 | 0.95 |  |
| Negative Emotional Response | 0.27 | 0.11 | 0.01* |  |
| *Step 4* |  |  |  | *0.08/0.14* |
| Gender | -0.06 | 0.10 | 0.57 |  |
| Education | -0.02 | 0.10 | 0.79 |  |
| Age | 0.01 | 0.10 | 0.90 |  |
| Hostile Attribution | -0.24 | 0.12 | 0.05 |  |
| Instrumental Attribution | -0.04 | 0.10 | 0.72 |  |
| Negative Emotional Response | 0.33 | 0.11 | 0.005** |  |
| Response Enactment (aggressive response) | -0.19 | 0.13 | 0.14 |  |

*β*, standardized regression coefficient.

**p* < .05, **p<0.01
